# Ustekinumab Intravenous Reinduction after Secondary Loss of Response in Patients with Crohn’s Disease

**DOI:** 10.1093/ibd/izaf163

**Published:** 2025-09-02

**Authors:** Stefan Schreiber, Scott D Lee, C Janneke van der Woude, Ignacio Marín-Jiménez, Douglas C Wolf, Elisabeth Schnoy, Bruce Salzberg, Christopher Busse, Maciej Nazar, Tony Ma, Stephan Borghorst, Christopher Gasink, Thomas Baker, Bridget Godwin, Omoniyi J Adedokun, Brian G Feagan, POWER Study Group, Scott Lee, Douglas Wolf, Bruce Salzberg, Elisabeth Schnoy, Stefan Schreiber, Jochen Klaus, Herbert Deppe, Anita Afzali, Thomas Krause, Yoram Bouhnik, Torsten Kucharzik, Oksana Shchukina, Renate Schmelz, Milan Lukas, Stephane Nancey, Byong Duk Ye, George DuVall, Stefanie Howaldt, Caprioli Flavio, Hyo-Jong Kim, Kamal Patel, Yoram Bouhnik, Pierre Blanc, Irina Blumenstein, Manreet Kaur, Jimmy Limdi, Annette Krummenerl, Peter Hasselblatt, Pilar Lopez Serrano, Ulf Helwig, Alessandro Armuzzi, Ana Echarri, Udayakumar Navaneethan, Britta Siegmund, Xavier Calvet Calvo, Parambir Dulai, Laurent Peyrin Biroulet, Wolfgang Reindl, Jeroen Jansen, Ignacio Marin Jimenez, Cristina Rodriquez Gutierrez, Raquel Vincente Lidon, Jessica Allegretti, Helga Török, Ji-Hye Park, Desirée Leemreis, Olga Fedorishina, Dawn Beaulieu, Sarah Glover, Charles Randall, Rodolfo Rocca, Mikael Lördal, William Holderman, Heba Iskandar, Laura Loy, Simone Saibeni, Maria Dolores Martin Arranz, Montserrat Rivero Tirado, Bincy Abraham, Robert Hirten, Tomas Vanasek, Guillaume Boughen, Pierre Desreumaux, Wolfgang Mohl, Taeoh Kim, Andrea van der Meulen, Pavel Makarchuk, Francis Farraye, Alexandra Kent

**Affiliations:** Department of Internal Medicine I, Christian-Albrechts-University and University Hospital Schleswig-Holstein, Kiel, Germany; Division of Gastroenterology, University of Washington, Seattle, WA, United States; Department of Gastroenterology and Hepatology, Erasmus University Medical Center, Rotterdam, The Netherlands; Gastroenterology Unit, Hospital Universitario Gregorio Marañón, IiSGM, Universidad Complutense, Madrid, Spain; Center for Crohn’s Disease & Ulcerative Colitis, Atlanta Gastroenterology Associates, Atlanta, GA, United States; Internal Medicine III, Gastroenterology, University Hospital of Augsburg, Augsburg, Germany; Atlanta Gastroenterology Specialists PC, IBD Center of Atlanta, Atlanta, GA, United States; Johnson & Johnson, Horsham, PA, United States; Johnson & Johnson, Warsaw, Poland; Johnson & Johnson, Horsham, PA, United States; Johnson & Johnson, Neuss, Germany; Johnson & Johnson, Horsham, PA, United States; Johnson & Johnson, Spring House, PA, United States; Johnson & Johnson, Spring House, PA, United States; Johnson & Johnson, Spring House, PA, United States; Division of Gastroenterology, Department of Medicine, University of Western Ontario, London, ON, Canada

**Keywords:** Crohn’s disease, intravenous reinduction, ustekinumab

## Abstract

**Background:**

The POWER study (NCT03782376) evaluated efficacy and safety of a single ustekinumab intravenous (IV) reinduction dose versus placebo under continued ustekinumab subcutaneous (SC) treatment in adult patients with moderately to severely active Crohn’s disease who demonstrated a secondary loss of response to ustekinumab every 8 weeks (q8w) maintenance therapy.

**Methods:**

Patients were randomly assigned 1:1 at Week 0 to ustekinumab IV reinduction (ustekinumab ∼6 mg/kg and SC placebo) or continuous maintenance (IV placebo and SC ustekinumab 90 mg q8w). Clinical and biomarker assessments occurred at Weeks 0, 8, 16, and 24 with optional ileocolonoscopy at Weeks 0 and16. The primary endpoint was clinical response (≥100-point decrease from baseline Crohn’s Disease Activity Index [CDAI] score or CDAI <150) at Week 16. Safety events were analyzed through Week 36 and serum samples were collected for pharmacokinetic analyses and anti-ustekinumab antibody detection.

**Results:**

Overall, 215 patients were randomized: 108 to the IV reinduction group and 107 to the SC group. In the IV reinduction group, 49.1% achieved clinical response at Week 16 versus 37.4% in the SC group (adjusted treatment difference 11.5% [95% CI: −1.5%, 24.5%; *P* = .089]). Proportions of patients with endoscopic remission and improvement, normalization of inflammatory biomarkers, and improvement in IBDQ score were greater in the IV reinduction group vs the SC group. No new safety signals were identified.

**Conclusions:**

Although the primary endpoint of clinical response was not met at Week 16, ustekinumab IV reinduction showed numerical improvements in objective endpoints including inflammatory biomarkers and endoscopic outcomes compared with SC maintenance therapy. Safety and immunogenicity results were consistent with the established profile of ustekinumab.

## Graphical abstract

**Graphical Abstract:**
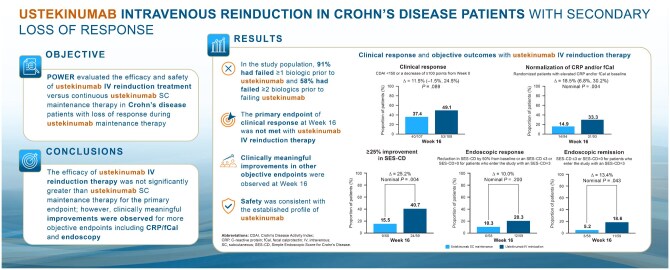

Key Messages **What is already known?**

Several biologic therapies, including ustekinumab, are approved for the treatment of Crohn’s disease; however, patients may lose response to biologics over time. Reinduction or dose interval shortening have been shown to help some patients regain response to these treatments.

**What is new here?**

POWER is the first randomized, placebo-controlled, double-blind trial to assess the efficacy and safety of ustekinumab IV reinduction in patients with Crohn's disease who have a secondary loss of response during ustekinumab SC maintenance therapy.

**How can this study help patient care?**

For certain patients with secondary loss of response, there may be some benefit of ustekinumab IV reinduction; however, this is not the standard treatment option.

## Introduction

Patients with Crohn’s disease require long-term treatment. However, some patients who initially respond to therapy may lose response, requiring dose intensification or switching to another agent.^1^^,^^2^ With biologic treatment, some patients with loss of response may receive reinduction or dose interval shortening, which can be effective in regaining response.^1^^,^^3–8^

For the anti-interleukin (IL)-12/IL-23 monoclonal antibody ustekinumab, approved maintenance dosing regimens, including recommendations for patients who lose response, vary by region. In the European Union, patients who lose response while receiving the recommended maintenance dose of subcutaneous (SC) ustekinumab 90 mg every 12 weeks (q12w) can receive shortened dose intervals of every 8 weeks (q8w). In the United States, the recommended maintenance dose interval is q8w. However, label guidance is lacking for patients who lose response while receiving q8w dosing.

Several retrospective, observational, or open-label studies evaluated the shortening of SC ustekinumab maintenance dose intervals to every 6 weeks (q6w) or every 4 weeks (q4w) and/or administering intravenous (IV) reinduction.^5^^,^^8–14^ However, none of these studies were blinded or randomized. Accordingly, robust data to determine the efficacy and safety of ustekinumab dose intensification are lacking.

Pharmacokinetic simulations based on an established population PK model in patients with Crohn’s disease^15^ predicted that a single IV reinduction dose of ustekinumab would provide substantial exposure to suppress the inflammatory burden in patients who lose response (Figure S1), thereby allowing them to return to standard maintenance dosing. Therefore, we conducted the POWER study to evaluate the efficacy and safety of a single ustekinumab IV reinduction dose versus continuous ustekinumab SC maintenance treatment in adult patients with moderately to severely active Crohn's disease who demonstrated a secondary loss of response to ustekinumab q8w maintenance treatment. Here we present the results of the POWER study with efficacy results through Week 24 and safety results through Week 36.

## Methods

### Study design

POWER was a multicenter, randomized, double-blind, placebo-controlled, 36-week, phase 3b study in adult patients (age 18 years or older) with moderately to severely active Crohn's disease diagnosed for at least 3 months, who initially responded to standard labeled ustekinumab induction therapy but subsequently lost response at any time after receiving at least 2 doses of SC q8w ustekinumab maintenance therapy (Figure S2). Loss of response was defined as a baseline Crohn’s Disease Activity Index^16^ (CDAI) score of ≥220 and ≤450 with at least one of the following: elevated C-reactive protein (CRP; >3.0 mg/L), elevated fecal calprotectin (fCal; >250 mg/kg), or endoscopy performed ≤3 months before baseline with evidence of active Crohn's disease (defined as one or more ulcerations in the ileum and/or colon). The study was conducted in 70 centers in 11 countries worldwide (Czech Republic, France, Germany, Italy, South Korea, the Netherlands, Russia, Spain, Sweden, the United Kingdom, and the United States). The study protocol was approved by the Institutional Review Board or Ethics Committee at each participating investigative center.

### Patients

Patients must have been receiving SC ustekinumab q8w maintenance therapy at enrollment. Patients were excluded from the study for: use of IV ustekinumab after the initial induction dose, gastrointestinal conditions that might require surgery or might preclude the use of the CDAI to assess the response to treatment, those with infections (including active tuberculosis) or a history of cancer, and any known history of shortened frequency of SC dose administration (<q8w) for a secondary loss of response where the patient did not, in the opinion of the treating physician, benefit from the dose interval shortening, had or suspected to have an abscess, any kind of bowel resection within 6 months or any other intra-abdominal surgery within 3 months before baseline, or a draining stoma or ostomy.

During the study, patients were permitted to receive oral 5-aminosalicylic acid compounds, the immunosuppressants methotrexate (MTX), azathioprine (AZA), and 6-mercaptopurine (6-MP), oral corticosteroids, and/or antibiotics for the treatment of Crohn's disease, provided they were receiving a stable dose for a protocol-specified period before baseline and maintained stable doses throughout the study. Per protocol, mandatory oral corticosteroids were tapered beginning at Week 8 in patients with a 70-point decrease from baseline in their CDAI score. All patients provided written informed consent. All authors approved the final manuscript.

### Randomization and masking

Eligible patients were randomly assigned to either the ustekinumab reinduction group (IV ustekinumab at a tiered weight-based dose of approximately 6 mg/kg and SC placebo) or the continuous maintenance group (IV placebo and SC ustekinumab 90 mg) at Week 0 in a 1:1 ratio by interactive web response system using permuted block randomization stratified at the study level by patients’ baseline CDAI score (≤300 or >300) and prior biologic failure (yes or no) at baseline. After Week 0, both groups received SC ustekinumab 90 mg at Weeks 8 and 16, and at Week 24 all patients returned to standard of care treatment (Figure S2).

### Procedures

Efficacy assessments were collected at baseline (Week 0) and at Weeks 8, 16, and 24 or at early termination. Assessments included CDAI score and Patient Reported Outcome (PRO-2; the CDAI components of the total number of liquid or very soft stools and the abdominal pain score in the prior 7 days, without weighting), inflammatory markers including serum CRP and fCal, and Inflammatory Bowel Disease Questionnaire (IBDQ) score (including IBDQ domains for bowel symptoms, systemic symptoms, social function, and emotional outcomes, with higher score indicating better outcomes). Endoscopic assessment of the intestinal mucosa consisted of evaluation of the presence or absence of mucosal ulcerations and the Simple Endoscopic Score for Crohn’s Disease (SES-CD). After a protocol amendment during the study (Protocol Amendment 4), endoscopy was not required, yet was made optional based upon patient consent at baseline and Week 16. The ileocolonoscopy was video recorded and all video endoscopies were assessed by a central facility that was blinded to the intervention group. To assess safety, adverse events, serious adverse events, infections, and serious infections, as well as changes in clinical laboratory test results, were evaluated. Serum samples were collected before and 60 minutes after study drug administration at baseline and before study drug administration at Weeks 8, 16, and 24, or at early termination for pharmacokinetic analyses and detection of anti-ustekinumab antibodies.

### Outcomes

The primary endpoint of this study was clinical response at Week 16, defined as a ≥ 100-point reduction from the baseline CDAI score or a CDAI score of <150 points. Major secondary endpoints included clinical response at Week 8; clinical remission (defined as a CDAI score of <150 points) at Week 8 and Week 16, and normalization of CRP (defined as CRP value ≤3 mg/L) and/or fCal (defined as ≤250 μg/g) concentrations at Week 16, among patients with an elevated CRP (>3 mg/L) and/or fCal (>250 µg/g) at baseline.

Clinical response, clinical remission, and normalization of CRP and/or fCal were also evaluated at Week 24. Change from baseline in the unweighted PRO-2 score was evaluated at Weeks 8, 16, and 24.

The proportion of patients with corticosteroid-free response (defined as a CDAI score decrease ≥100 from baseline or a CDAI score of <150 points and no steroid use for at least 30 days prior to timepoint) at Week 24, the proportion of patients with corticosteroid-free response at Week 24 among patients who were receiving corticosteroids at baseline, and the proportion of patients with corticosteroid free remission (defined as a CDAI score <150 among patients who were receiving corticosteroids at baseline) at Week 24 were also evaluated.

Endoscopy endpoints at Week 16 included endoscopic response (reduction of ≥50% from baseline in SES-CD or SES-CD score ≤3), endoscopic remission (SES-CD score ≤3), change from baseline in SES-CD score, and the proportion of patients with a ≥25% improvement from baseline in SES-CD score.

Health-related quality of life was evaluated using the change from baseline in the IBDQ score (including IBDQ domains) at Week 16 and the proportion of patients with IBDQ remission (IBDQ score ≥170) at Week 16.

### Statistical analyses

Assuming a 60% clinical response rate at Week 16 in the ustekinumab IV reinduction group and 40% in ustekinumab SC maintenance group, it was determined that 100 patients per treatment group would yield an overall power greater than 80%, at a significance level of 0.05 (2-sided, Mantel-Haenszel test). We planned to randomize and treat approximately 200 patients.

Efficacy analyses for the primary and other endpoints were provided for the full analysis set, defined as all randomized patients. For the primary, secondary, and other efficacy endpoints, the proportions of patients who reached each endpoint were compared between treatment groups using the two-sided Cochran-Mantel-Haenszel χ^2^ test at a significance level of 0.05, with adjustment for randomization stratification factors. Continuous variables were compared between treatment groups using an analysis of covariance on van der Waerden normal scores with baseline value and randomization stratification factors as covariates. Patients who had a prohibited Crohn’s disease-related surgery, had prohibited concomitant medication changes, or discontinued study agent due to lack of efficacy or due to an adverse event of worsening Crohn’s disease, were deemed to have had treatment failure and to have not reached dichotomous efficacy endpoints from the time the treatment failure occurred.

For continuous efficacy endpoints, the baseline value was carried forward from the time the treatment failure occurred. Missing values were imputed as not having reached the endpoint for dichotomous outcomes or using the last observation carried forward approach for continuous outcomes.

Safety analyses were performed for the full analysis population who received at least 1 administration of study drug and were analyzed according to the actual treatment received. This study is registered with ClinicalTrials.gov (NCT03782376).

### Role of funding source

The study funder was responsible for the study design; the collection, analysis, and interpretation of data; the writing of the report; and the decision to submit the paper for publication.

## Results

POWER was conducted at 70 centers between 15 January 2019 and 10 January 2023. A total of 215 patients were randomized; 108 patients were assigned and treated in the ustekinumab IV reinduction group, and 107 were assigned and treated in the ustekinumab SC maintenance group. A total of 12 patients (*n* = 6 in each group) who had previously benefited from dose optimization (a SC maintenance interval <8 weeks) were included in the study. The median time (interquartile range) since the last optimized dose was 47.60 (28.0, 72.0) weeks for the IV reinduction group and 23.65 (23.1, 93.9) weeks for the ustekinumab SC maintenance group. Through Week 16, 8 patients (7.4%) in the ustekinumab IV group and 15 patients (14.0%) in the ustekinumab SC group discontinued the study. At Week 36, 82 patients (75.9%) in the ustekinumab IV reinduction group and 77 patients (72.0%) in the ustekinumab SC maintenance group completed the final follow-up (Figure S3).

Demographic characteristics were similar between treatment groups; the overall mean age was 40.9 years, and approximately 58% of patients were female (Table 1). Baseline Crohn’s disease characteristics were generally reflective of a population with moderate to severely active Crohn’s disease. Crohn’s disease medication history (corticosteroids and/or MTX/AZA/6-MP) was balanced across treatment groups at baseline. Overall, 63 (58.3%) and 62 (57.9%) patients had endoscopies available at baseline in the ustekinumab IV reinduction group and the ustekinumab SC maintenance group, respectively. Of those, 4 patients in each group had SES-CD <3 at baseline and were not included in the endoscopic endpoint analyses. Baseline demographics and disease characteristics among patients with endoscopy were similar to those of the overall population (Table S2).

**Table 1.** Baseline demographics and disease characteristics (overall population).

	Ustekinumab SC maintenance	Ustekinumab IV reinduction	Combined
*N*	107	108	215
Age, years			
Mean (SD)	40.0 (13.07)	41.8 (13.63)	40.9 (13.36)
Sex			
Female, *n* (%)	62 (57.9)	62 (57.4)	124 (57.7)
Age at diagnosis			
Mean (SD)	25.9 (13.43)	27.2 (13.18)	26.5 (13.29)
Crohn’s Disease duration, years			
Mean (SD)	14.1 (10.14)	14.7 (10.84)	14.4 (10.48)
Involved GI areas, *n* (%)			
Ileum only	28 (26.2)	29 (26.9)	57 (26.5)
Colon only	22 (20.6)	24 (22.2)	46 (21.4)
Ileum and colon	55 (51.4)	55 (50.9)	110 (51.2)
Proximal	13 (12.1)	20 (18.5)	33 (15.3)
Perianal	34 (31.8)	33 (30.6)	67 (31.2)
Patients with MTX/AZA/6-MP or corticosteroid use at baseline	39 (36.4)	30 (27.8)	69 (32.1)
Immunomodulators (MTX/AZA/6-MP)	19 (17.8)	16 (14.8)	35 (16.3)
Corticosteroids (including budesonide)	22 (20.6)	16 (14.8)	38 (17.7)
Corticosteroids (excluding budesonide)	11 (10.3)	9 (8.3)	20 (9.3)
Patients with inadequate response to corticosteroids and MTX/AZA/6-MP	40 (37.4)	46 (42.6)	86 (40.0)
Patients with inadequate response to corticosteroids only	13 (12.1)	18 (16.7)	31 (14.4)
Patients with inadequate response to MTX/AZA/6-MP only	33 (30.8)	26 (24.1)	59 (27.4)
History of inadequate response or intolerance to biologics before ustekinumab,^a^ ^,^ ^b^ *n* (%)			
No history of inadequate response or intolerance (biologic naive or experienced)	8 (7.5)	12 (11.1)	20 (9.3)
≥1 biologic (anti-TNF agent or vedolizumab) before ustekinumab^a^ ^,^ ^b^	99 (92.5)	96 (88.9)	195 (90.7)
≥2 biologics (≥1 anti-TNF agents ± vedolizumab) before ustekinumab^a^ ^,^ ^b^	62 (57.9)	63 (58.3)	125 (58.1)
≥3 biologics (1 anti-TNF agent ± vedolizumab before ustekinumab^a^ ^,^ ^b^	27 (25.2)	28 (25.9)	55 (25.6)
≥1 anti-TNF + vedolizumab^c^ before ustekinumab^a^ ^,^ ^b^	38 (35.5)	33 (30.6)	71 (33.0)
CDAI score			
Mean (SD)	289.9 (55.84)	288.6 (55.47)	289.2 (55.53)
PRO-2 (without weighting)^d^			
Median	47.0	47.0	NA
IQ range	(35.0; 58.0)	(35.0; 62.0)	
CRP (mg/L)			
Median	5.1	5.7	5.6
IQ range	(2.6; 12.6)	(2.5; 14.6)	(2.6; 13.7)
fCal (mg/kg)			
Median	706.0	449.5	515.0
IQ range	(197.0; 1849.0)	(169.5; 1448.0)	(173.0; 1710.0)
IBDQ score^e^ (32-224)			
*N*	105	107	212
Mean (SD)	122.8 (33.45)	118.4 (27.89)	120.6 (30.77)
SES-CD score^f^			
*N*	62	63	125
Mean (SD)	10.7 (7.31)	9.8 (7.01)	10.2 (7.15)
Endoscopy available at baseline,^g^ *n* (%)	62 (57.9)	63 (58.3)	125 (58.1)

Abbreviations: 6‑MP = 6‑mercaptopurine; AZA = azathioprine; CDAI = Crohn’s Disease Activity Index; CRP = C-reactive protein; fCal = fecal calprotectin; GI = gastrointestinal; IBDQ = Inflammatory Bowel Disease Questionnaire; IQ = interquartile; IV = intravenous; MTX = methotrexate; PRO-2 = Patient Reported Outcome; SC = subcutaneous; SD = standard deviation; SES-CD = Simple Endoscopic Score for Crohn’s Disease; TNF = tumor necrosis factor.

a Patients with a history inadequate response or intolerance to biologic treatment characterized by primary nonresponse, secondary nonresponse, or intolerance.

b Anti-TNFs were adalimumab, infliximab, and certolizumab pegol.

c Patients with inadequate response or intolerance to at least 2 mechanisms of action before receiving ustekinumab, regardless of the number of anti-TNFs.

d Sum of the number of stools and the abdominal pain scores in the previous 7 days.

e The IBDQ total scores were used, ranging from 32 to 224.

f Endoscopy was an optional procedure during the study.

g Overall, 4 patients in each group had SES-CD < 3 at baseline and were not included in the endoscopic endpoint analyses.

**Figure 1.**
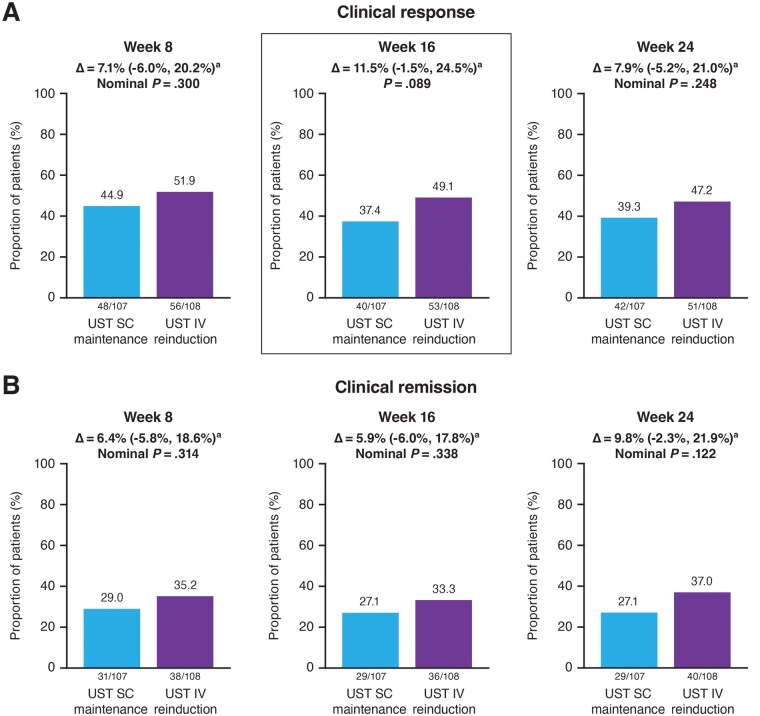
(A) Clinical response and (B) Clinical remission at Weeks 8, 16, and 24.^b^Abbreviations: CD = Crohn’s disease; CDAI = Crohn’s Disease Activity Index; CI = confidence interval; IV = intravenous; SC = subcutaneous; UST = ustekinumab The primary endpoint of the study was clinical response at Week 16. As the primary endpoint was not met, all p values for other comparisons between treatment groups should be considered nominal. Clinical response is defined by CDAI < 150 or decrease of ≥ 100 points from Week 0. Clinical remission is defined as CDAI score < 150. a2‑sided Cochran–Mantel–Haenszel–chi‑square test, stratified by baseline CDAI score (≤ 300 or > 300) and prior biologic failure status at baseline (yes or no) at a significance level of 0.05; 95% CI for adjusted treatment difference; the 95% CIs were based on the Wald statistic with Mantel–Haenszel weight. bPatients who had insufficient data at the designated analysis timepoint or a prohibited CD‑related surgery, prohibited concomitant medication changes, or discontinued study agent due to lack of efficacy or due to an adverse event indicated to be of worsening CD prior to the designated analysis timepoint were not considered to have achieved clinical response/remission (regardless of CDAI score).

Overall, 49.1% of patients in the ustekinumab IV reinduction group achieved clinical response at Week 16 compared with 37.4% in the ustekinumab SC maintenance group; absolute difference 11.5% (95% CI: −1.5%, 24.5%, *P* = .089; Figure 1A). The primary endpoint was not met; therefore, all *P*-values for the major secondary endpoints were considered nominal. Proportions of patients in clinical response at Week 8 were slightly greater in both groups than those at Week 16; proportions at Week 24 were similar to those at Week 16 (Figure 1A).

Proportions of patients in clinical remission for each treatment group were numerically higher in the IV group at all visits, with a difference of almost 10% at Week 24 (all nominal *P*-values >.05; Figure 1B). At Weeks 8, 16, and 24, patients in the ustekinumab IV reinduction group had numerically greater change from baseline in the unweighted PRO-2 than patients in the ustekinumab SC maintenance group at all visits (Table S1). Mean and median changes from baseline in CDAI scores (post hoc analysis) are summarized in Table S1.

**Figure 2.**
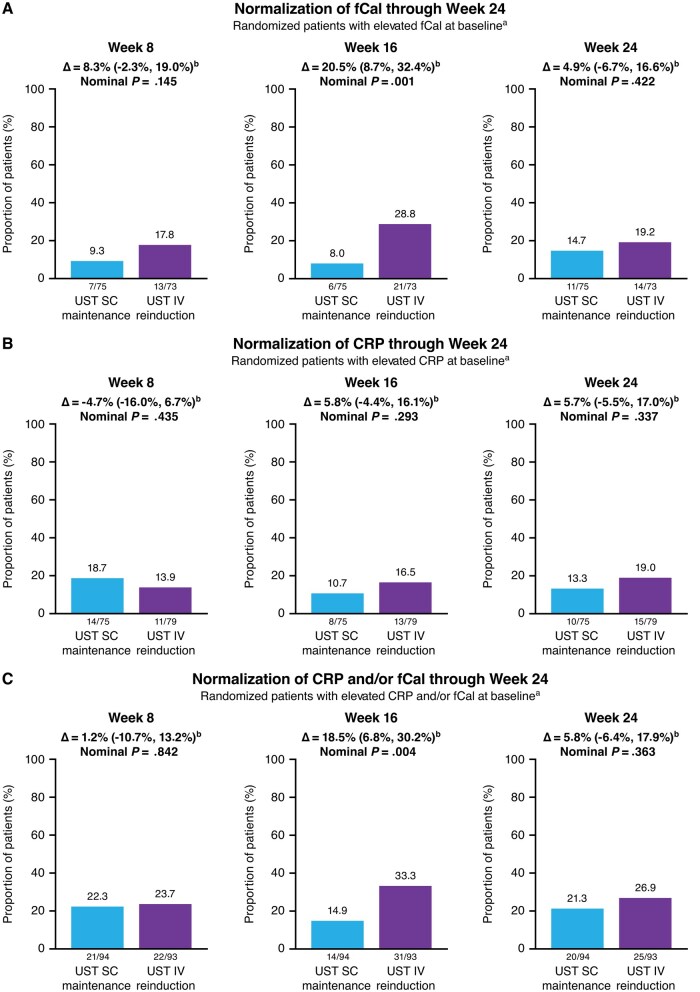
Normalization of (A) fCal and (B) CRP at Week 8, Week 16, and Week 24 among randomized patients with elevated levels at baseline. (C) Normalization of CRP and/or fCal through Week 24 among randomized patients with elevated levels at baseline.Abbreviations: CD = Crohn’s disease; CRP = C‑reactive protein; fCal = fecal calprotectin; IV = intravenous; SC = subcutaneous; UST = ustekinumab Normalized CRP was defined as a CRP ≤ 3 mg/L and normalized fCal was defined as fCal ≤ 250 μg/g. As the primary endpoint was not met, all p values for other comparisons between treatment groups should be considered nominal. aPatients who had insufficient data at the designated analysis timepoint or a prohibited CD‑related surgery, prohibited concomitant medication changes, or discontinued study agent due to lack of efficacy or due to an adverse event indicated to be of worsening CD prior to the designated analysis timepoint were not considered to have achieved biomarker response. bThe confidence intervals were based on the Wald statistic with Mantel–Haenszel weight.

Among patients with fCal concentrations >250 µg/g at baseline, the proportions of patients with normalized fCal concentrations (≤250 µg/g) at Week 8 were 17.8% in the ustekinumab IV reinduction group and 9.3% in the ustekinumab SC maintenance group (Figure 2A). At Week 16, the proportion of patients attaining normalized CRP increased to 28.8% in the IV reinduction group and decreased to 8.0% in the SC maintenance group. At Week 24, these proportions became more similar, with 19.2% in the IV reinduction group and 14.7% in the SC maintenance group.

Among patients with CRP concentrations >3 mg/L at baseline, the proportions of patients with normalized CRP (≤ 3 mg/L) evolved from 13.9% in the ustekinumab IV reinduction group and 18.7% in the ustekinumab SC maintenance group at Week 8 to 16.5% and 10.7%, respectively, at Week 16, and to 19.0% and 13.3%, respectively, at Week 24 (Figure 2B).

Among patients with elevated CRP and/or fCal concentrations at baseline, 23.7% of patients in the ustekinumab IV reinduction group and 22.3% ustekinumab SC maintenance group had normalized CRP and/or fCal at Week 8. The proportions of patients with normalized CRP and/or fCal decreased in the ustekinumab SC maintenance group (14.9%) and increased in the ustekinumab IV reinduction group (33.3%) at Week 16, but proportions were more similar between groups at Week 24 (21.3% and 26.9%, respectively; Figure 2C).

**Figure 3.**
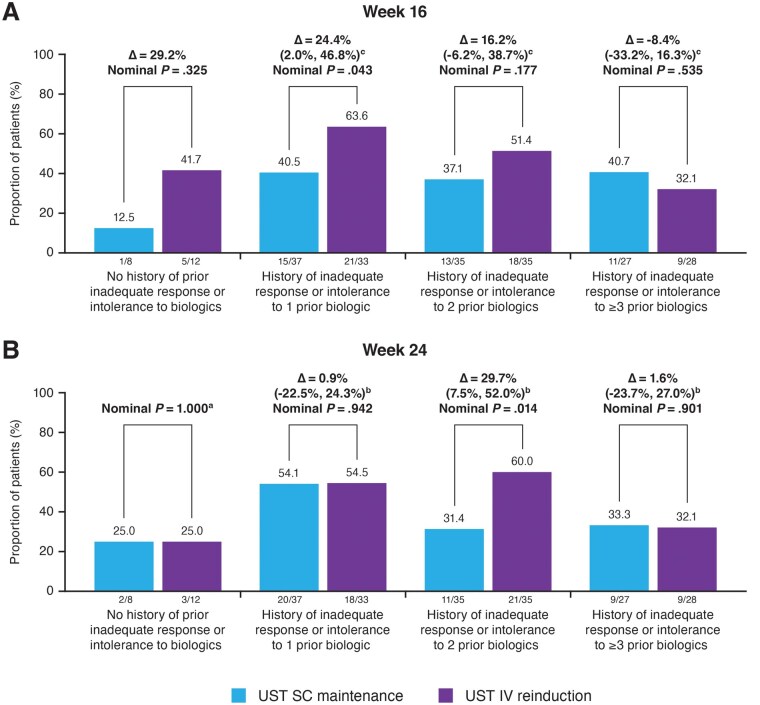
Clinical response at (A) Week 16 and (B) Week 24 based on number of biologics with inadequate response or intolerance before starting ustekinumab.^a,b^Abbreviations: CD = Crohn’s disease; CDAI = Crohn’s Disease Activity Index; IV = intravenous; SC = subcutaneous; UST = ustekinumab As the primary endpoint was not met, all p values for other comparisons between treatment groups should be considered nominal. aClinical response is defined by CDAI < 150 or decrease of ≥ 100 points from Week 0. bPatients who had insufficient data at the designated analysis timepoint or a prohibited CD‑related surgery, prohibited concomitant medication changes, or discontinued study agent due to lack of efficacy or due to an adverse event indicated to be of worsening CD prior to the designated analysis timepoint were not considered to have achieved clinical response/remission (regardless of CDAI score). cThe confidence intervals were based on the Wald statistic with Mantel–Haenszel weight.

At Week 16, among patients with no prior history of inadequate response or intolerance to biologics before ustekinumab, more patients in the ustekinumab IV reinduction group than in the ustekinumab SC maintenance group achieved clinical response (41.7% of patients in the IV reinduction group vs 12.5% in the SC maintenance group). Proportions of patients in clinical response at Week 16 among patients with a history of inadequate response or intolerance to just 1 prior biologic before ustekinumab (63.6% versus 40.5% for the ustekinumab IV reinduction and SC maintenance group, respectively) were numerically greater than those with 2 prior biologics in both groups (51.4% versus 37.1%; Figure 3A). Among more refractory patients with a history of inadequate response or intolerance to ≥3 prior biologics, more patients in the ustekinumab SC maintenance group (40.7%) achieved clinical response at Week 16 compared with those in the IV reinduction group (32.1%). At Week 24, the proportions of patients who achieved clinical response were similar in both treatment groups for patients who had no history of biologics before ustekinumab (25.0% and 25.0%, respectively), history of inadequate response or intolerance to 1 previous biologic (54.1% and 54.5%), and history of inadequate response or intolerance to ≥3 previous biologics (33.3% and 32.1% for the SC and IV groups, respectively). Among patients with a history of inadequate response or intolerance to 2 previous biologics, the proportion of patients who achieved a clinical response was numerically higher in the ustekinumab IV reinduction group compared with the SC maintenance group (60.0% versus 31.4%, respectively; Figure 3B). 

**Figure 4.**
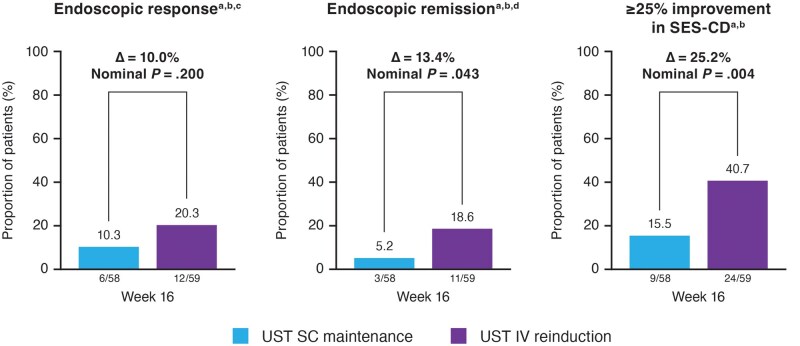
Endoscopic endpoints (≥25% improvement in SES-CD; endoscopic response, or endoscopic remission) at Week 16.Endoscopy was optional and available for a certain proportion of patients only. Abbreviations: IV, intravenous; SC, subcutaneous; SES‑CD, Simple Endoscopic Score for Crohn’s Disease; UST, ustekinumab As the primary endpoint was not met, all p values for other comparisons between treatment groups should be considered nominal. ^a^Patients who had a prohibited Crohn’s disease‑related surgery, had prohibited concomitant medication changes, or discontinued study agent due to lack of efficacy or due to an adverse event indicated to be of worsening Crohn’s disease prior to the designated analysis timepoint are considered not to be in SES‑CD improvement, endoscopic response, or remission, regardless of their SES‑CD score. ^b^Patients who had insufficient data to calculate the SES‑CD score at the designated analysis timepoint are considered not to be in SES‑CD improvement. ^c^Endoscopic response is defined as a reduction in SES‑CD score by 50% from baseline or SES‑CD score ≤ 3 or SES‑CD = 0 for patients who enter the study with an SES‑CD = 3. ^d^Endoscopic remission is defined as an SES‑CD score ≤3 or SES‑CD = 0 for patients who enter the study with an SES‑CD = 3.

Among the patients who consented to endoscopy and had a SES-CD score ≥3 at baseline, 20.3% of patients in the ustekinumab IV reinduction group versus 10.3% of in the ustekinumab SC maintenance group achieved endoscopic response at Week 16, and 18.6% versus 5.2% achieved clinical remission at Week 16, respectively (Figure 4). Similarly, 40.7% of patients in the ustekinumab IV reinduction group and 15.5% of patients in the ustekinumab SC maintenance group had ≥25% improvement from baseline in SES-CD score at Week 16. Median (interquartile range) change from baseline in SES-CD score at Week 16 was −1.0 (−4.0; 0.0) in the ustekinumab IV reinduction group versus 0.0 (−1.0; 0.0) in the ustekinumab SC maintenance group. 

At Week 16, the proportion of patients who achieved IBDQ response (≥16-point improvement from baseline IBDQ score) and who achieved IBDQ remission (IBDQ score ≥170) was numerically higher in the ustekinumab IV reinduction group compared with the ustekinumab SC maintenance group (Figure S4).

Patients with a disease duration of <5 years, patients with both ileum and colon involvement, patients with elevated baseline CRP or fCal, patients with history of inadequate response or intolerance to 1 previous biologic, and patients with a prior perianal Crohn's disease-related surgery were more likely to achieve clinical response at Week 16 with ustekinumab IV reinduction than ustekinumab SC maintenance (Figure S5).

Serum ustekinumab concentration data were obtained from 215 patients with ≥1 blood sample collected (ustekinumab IV reinduction, *n* = 108; SC maintenance, *n* = 107). The baseline median serum ustekinumab concentrations were similar for both groups (1.30 μg/mL and 1.26 μg/mL, respectively, for IV and SC groups). Following IV administration of ∼6 mg/kg ustekinumab, the median concentration post-dose increased to 105.6 μg/mL. In contrast, the median concentration in the ustekinumab SC maintenance group was 1.5 μg/mL. During the treatment period, the median pre-administration (steady-state trough) ustekinumab concentrations were consistently numerically higher in the IV reinduction group compared with the SC maintenance group (Figure S6) remaining 2-fold higher at Week 16 and becoming similar at Week 24. Ustekinumab serum concentration at baseline did not predict clinical response at Week 16. (Figure S7). A numerically greater proportion of patients with ustekinumab serum trough levels ≥1.3 µg/mL achieved endoscopic remission at Week 16 in the IV versus the SC group (Figure S8A) and a numerically greater proportion achieved endoscopic remission in the IV group versus the SC group in all ustekinumab trough concentration quartiles at Week 16 (Figure S8B). The incidence of ustekinumab anti-drug antibodies through Week 24 was low; 2 (1.9%) patients in the ustekinumab SC group and none in the ustekinumab IV reinduction group. Of the two patients with anti-drug antibodies, one had anti-drug antibodies at baseline.

Through Week 36, the mean duration of safety follow-up was 32.6 weeks in the ustekinumab IV reinduction group and 30.3 weeks in the ustekinumab SC maintenance group. Similar proportions of patients reported treatment-emergent adverse events through Week 36, 70.4% in the ustekinumab IV reinduction group and 72.9% in the ustekinumab SC maintenance group (Table 2). The proportions of patients with treatment-emergent serious adverse events (SAEs) through Week 36 were 8.3% in the ustekinumab IV reinduction group and 12.1% in the ustekinumab SC maintenance group, with no COVID-19-related SAEs.

**Table 2.** Treatment-emergent adverse events through Week 36; safety analysis set.

	Ustekinumab SC maintenance	Ustekinumab IV reinduction
*N*	107	108
Duration of follow-up (weeks), mean	30.3	32.6
Number of study agent administrations, mean	3.7	3.8
Patients with 1 or more: *n* (%)		
Adverse events (AEs)	78 (72.9)	76 (70.4)
COVID-19 related AEs	17 (15.9)	12 (11.1)
AEs leading to discontinuation of study agent	10 (9.3)	9 (8.3)
Infusion-related^a^ AEs	1 (0.9)	1 (0.9)
Serious AEs (SAEs)	13 (12.1)	9 (8.3)
COVID-19 related SAEs	0	0
Infections	32 (29.9)	36 (33.3)
Serious infections	2 (1.9)	2 (1.9)
Injection site reactions	2 (1.9)	2 (1.9)
Malignancies	1 (0.9)	0
Deaths^b^	0	1 (0.9)

Infusion-related AEs and injection site reactions include all AEs/reactions for both active and placebo treatments.

Abbreviations: AE = adverse event; IV = ustekinumab; SAE = serious adverse event; SC = subcutaneous.

a Adverse events during or within 1 hour of a study agent infusion.

b Adverse events leading to death are based on adverse event outcome of fatal.

Infections were reported in 33.3% of the patients in the ustekinumab IV reinduction group and in 29.9% of the patients in the ustekinumab SC maintenance group, with serious infections reported in 2 patients (1.9%) in each treatment group.

The proportion of patients with adverse events leading to discontinuation through Week 36 was similar between treatment groups, 8.3% in the ustekinumab IV reinduction group and 9.3% in the ustekinumab SC maintenance group. There were no infusion reactions or allergic reactions to ustekinumab reported in either of the patients with ustekinumab anti-drug antibodies, including the one patient with anti-drug antibodies at baseline.

No opportunistic infections were reported. Through Week 36, one malignancy (malignant melanoma) was reported in the ustekinumab SC maintenance group. Through Week 36, one death was reported in a 62-year-old man in the ustekinumab IV reinduction group with a history of psoriasis, obesity, hypertension, familial early coronary artery disease, and tobacco use. The cause of death was listed as acute myocardial infarction and was deemed unrelated to study treatment.

## Discussion

Robust evidence is needed to inform physicians in the management of patients who have a loss of response to Crohn's disease treatments. For ustekinumab, dose intensification occurs in usual clinical practice; however, the evidence supporting this approach is limited to uncontrolled observational or retrospective studies.^6^^,^^8^ POWER is the first randomized, controlled, double-blind trial to assess the efficacy and safety of ustekinumab IV reinduction in patients with loss of response during ustekinumab SC maintenance therapy.

The CDAI-based primary endpoint of the study at Week 16 was not met; the observed difference between the treatment groups in the proportion of patients who were in CDAI-clinical response was not statistically significant. The CDAI is a good tool for assessing the efficacy of active treatment versus placebo,^17^ but may be inferior to objective markers when assessing the efficacy of treatment optimization. Furthermore, the CDAI is a subjective outcome, dominated by symptom-based items. Also, this study was relatively underpowered to detect a subtle clinical difference. In this regard, it is notable that many of the objective secondary outcomes were consistent with a benefit of IV reinduction. Symptomatic outcomes, which are important to both patients and physicians, were greater in patients who received IV reinduction compared with those who received SC maintenance. Additionally, patients who received IV reinduction had clinically meaningful improvements in objective endpoints, including inflammatory biomarkers and endoscopic outcomes.

Overall, the study population was highly refractory to treatment, with a mean disease duration of 14.4 years, more than half of patients having a history of an inadequate response or intolerance to 2 or more biologics before receiving ustekinumab, and only 9.3% of patients having no such history. The treatment effect of ustekinumab reinduction was not consistent for subgroups of patients based on history of inadequate response or intolerance to biologics. Biologic-naive patients and those with 1 or 2 prior biologics before ustekinumab appeared to have a benefit from IV reinduction, as did patients with a high inflammatory burden, whereas the improvement with IV reinduction was most modest in patients with a history of 3 or more prior biologics before ustekinumab. These results should be interpreted carefully due to the small number of patients. Given that this highly refractory subgroup comprised one quarter of the overall study population, it is not surprising that only an 11% benefit was observed for the ustekinumab IV reinduction strategy relative to SC maintenance. These results suggest that these more refractory patients may have benefit in switching to an alternative therapy, although these patients may be the least likely to respond to a new therapy.^18^^,^^19^ Interestingly, more than one third of patients were able to regain response with continued q8w maintenance therapy and the level of response after IV reinduction was similar at Week 24, demonstrating that patients were able to maintain response. However, it is also noteworthy that patients with baseline characteristics indicating greater Crohn's disease severity according to objective measures, such as greater baseline CRP or fecal calprotectin levels, appeared to benefit more from ustekinumab IV reinduction than those with lower disease severity.

Low baseline serum ustekinumab concentrations did not seem to predict who would be more likely to respond to intensified dosing, as would be expected with typical therapeutic drug monitoring approaches. Almost half of patients had baseline concentrations below 1.3 µg/mL, a level previously shown to be associated with clinical efficacy.^20^ Across biologics, studies have shown an association between drug levels and efficacy;^20^^,^^21^ however, it is not always clear if increased levels are the causative factor versus simply an association seen retrospectively. Notably, ustekinumab clearance has been reported to be more strongly associated with efficacy outcomes than trough concentrations.^22^^,^^23^ This study provided the ability to prospectively determine how baseline drug levels might impact the likelihood that dose intensification would provide benefit. If drug concentrations were deterministic, it would be expected that patients with the lowest baseline ustekinumab levels would be more likely to benefit from increased exposure from the IV reinduction. However, in this study, patients with lower baseline levels were not more likely to respond to dose intensification with IV reinduction compared with patients with higher baseline ustekinumab levels, indicating that levels at the time of loss of response did not predict who was more likely to respond to intensification with IV reinduction.

Post-baseline, a positive exposure-response relationship was observed in this study, as higher Week-16 ustekinumab serum concentrations were associated with numerically greater proportions of patients achieving endoscopic remission. This trend was more apparent in patients who received ustekinumab IV reinduction than SC maintenance.

In three previous real-world studies, patients showed benefit from ustekinumab IV reinduction, with clinical remission rates from 31% to 49%, although these studies were not blinded, did not have a comparator without reinduction, and included patients who had received varied maintenance dosing intervals before and after IV reinduction.^7^^,^^9^^,^^10^ In our study, the proportion of patients in clinical remission after IV reinduction was 33.3% at Week 16 and 37.0% at Week 24. The strength of our study was the randomized, double-blind, placebo-controlled design and consistent maintenance dosing prior to and after IV reinduction. In the prospective, randomized, double-blind, placebo-controlled REScUE study, low proportions of patients (16%-17%) achieved the primary endpoint of steroid-free clinical remission at Week 48 after a single IV re-induction with ustekinumab.^24^ Of note, no appreciable difference between the q8w maintenance group (16%) and the q4w maintenance group (17%) was observed. Differences between studies in patient selection, endpoint definitions, and timing of analyses should also be taken into consideration when interpreting the results.

There were some limitations to this study. First, the primary endpoint was based upon the CDAI, which has previously noted limitations. Additionally, with a sample size of 200 patients, the study was designed to detect a 20% difference, which was predicted based on subgroup data in previous studies. However, the study was relatively underpowered to detect the actual treatment difference observed (11.5%). Objective measures such as endoscopic or inflammatory biomarker outcomes may be better for assessing the efficacy of dose intensification strategies in highly treatment-experienced patient populations. However, in this trial, only 45% of patients underwent baseline and Week-16 endoscopic assessments, although the patients undergoing endoscopic assessments had similar baseline characteristics as the complete study population (Table S2), it is possible that these results may be influenced by a potential selection bias of those patients consenting to the endoscopic assessment. Additionally, with the final assessment at Week 24, it cannot be determined if patients may need periodic IV reinduction and/or other modifications such as q4w maintenance to both enhance and maintain response. Importantly, the study was conducted during the COVID-19 pandemic, which may have limited the ability for investigators to obtain endoscopic assessments.

In conclusion, while the primary endpoint of the study was not met, some patients with secondary loss of response to ustekinumab SC maintenance treatment who received ustekinumab IV reinduction had some clinically meaningful improvements in objective measures of disease activity, symptomatic outcomes, and quality of life. Safety and immunogenicity results were consistent with the established profile of ustekinumab, demonstrating that ustekinumab IV reinduction may be a safe and effective therapy for some patients who have lost response to standard ustekinumab SC maintenance therapy.

## Supplementary Material

izaf163_Supplementary_Data

## Data Availability

The data sharing policy of Johnson & Johnson is available at https://innovativemedicine.jnj.com/our-innovation/clinical-trials/transparency. As noted on this site, requests for access to the study data can be submitted through Yale Open Data Access (YODA) Project site at http://yoda.yale.edu.
