# Supplementary material for: Ustekinumab Intravenous Reinduction after Secondary Loss of Response in Patients with Crohn’s Disease
Source: Inflamm Bowel Dis. 2025 Sep 2;31(12):3286–97. doi: 10.1093/ibd/izaf163 (PMC12688074; doi:10.1093/ibd/izaf163)
Supplement: izaf163_Supplementary_Data [file izaf163_supplementary_data.zip › POWER Ms_R2_Updated Supplement_clean.docx]

**SUPPLEMENTARY MATERIALS**

**Page**

Supplemental Table 1. Change from Baseline in CDAI Score and PRO-2…………………... 39

Supplemental Table 2. Baseline Demographics and Disease Characteristics Among Patients

with Endoscopy (Baseline SES-CD Score ≥3) ………………….…………………………… 40

Supplemental Figure 1. Pharmacokinetic modelling of ~6mg/kg IV Reinduction Versus SC

90 mg q4w injections………………………..………………………………………………… 42

Supplemental Figure 2. POWER Study Design………………………...…………………….. 43

Supplemental Figure 3. CONSORT Diagram………………………………………………… 44

Supplemental Figure 4. IBDQ Response and Remission at Week 16………………………… 45

Supplemental Figure 5. Clinical Response at Week 16 by Baseline Characteristics ………… 46

Supplemental Figure 6. Median Serum Ustekinumab Concentrations……………………….. 47

Supplemental Figure 7. Patients in Clinical Response at Week 16 by Ustekinumab Serum

Through Concentration Quartile Groups at Baseline……………………….……………….... 48

Supplemental Figure 8. Endoscopic Remission at Week 16 by (A) Ustekinumab Serum

Trough Concentration and (B) Ustekinumab Trough Quartiles….…….….……………..…… 49

**Supplemental Table 1. Change from Baseline in CDAI Score and PRO-2**

|  | **Ustekinumab SC**  **Maintenance** | **Ustekinumab IV**  **Reinduction** |
| --- | --- | --- |
| N | 107 | 108 |
| **CDAI^a^** |  |  |
| Week 8^b,c^ |  |  |
| Mean (SD) | -79.6 (94.11) | -99.8 (101.03) |
| Median (IQR) | -80.0 (-136.0; -9.0) | -102.5 (-168.5; -31.5) |
| Nominal p-value | -- | 0.112 |
| Week 16^b,c^ |  |  |
| Mean (SD) | -80.4 (91.76) | -99.3 (105.49) |
| Median (IQR) | -67.0 (-144.0; 0.0) | -94.5 (-177.5; -36.0) |
| Nominal p-value | -- | 0.113 |
| Week 24^b.c^ |  |  |
| Mean (SD) | -83.9 (95.99) | -104.4 (104.56) |
| Median (IQR) | -72.0 (-150.0; 0.0) | -112.5 (-179.5; -31.0) |
| Nominal p-value | -- | 0.095 |
|  |  |  |
| **PRO-2** |  |  |
| Week 8^b,c^ |  |  |
| Mean (SD) | -12.8 (20.87) | -19.8 (19.73) |
| Median (IQR) | -10.0 (-24.0; 0.0) | -16.0 (-30.0; -6.5) |
| Nominal p-value | -- | 0.004 |
| Week 16^b,c^ |  |  |
| Mean (SD) | -12.5 (20.78) | -19.2 (22.62) |
| Median | -10.0 (-24.0; 0.0) | -15.5 (-30.0; -0.5) |
| Nominal p-value | -- | 0.017 |
| Week 24^b,c^ |  |  |
| N |  |  |
| Mean (SD) | -13.5 (20.68) | -18.6 (21.42) |
| Median (IQR) | -11.0 (-23.0; 0.0) | -18.0 (-27.0; -3.5) |
| Nominal p-value | -- | 0.029 |

CDAI, Crohn’s Disease Activity Index; IQR, interquartile range; IV, intravenous; PRO-2, Patient Reported Outcome-2; SC, subcutaneous; SD, standard deviation

^a^ Summary of changes from baseline CDAI scores was performed as a post hoc analysis.

^b^ Patients who had a prohibited Crohn's disease-related surgery, discontinued due to lack of efficacy or due to an adverse event indicated to be of worsening Crohn's disease, or had prohibited concomitant medication changes prior to the designated analysis time point had their baseline value carried forward.

^c^ Patients who had insufficient data to calculate the CDAI score at the designated analysis timepoint had their last value carried forward.

**Supplemental Table 2. Baseline Demographics and Disease Characteristics Among Patients with Endoscopy (Baseline SES-CD Score ≥3)**

|  | **Ustekinumab SC Maintenance** | **Ustekinumab IV Reinduction** | **Combined** |
| --- | --- | --- | --- |
| Patients with SES-CD Score ≥3 at baseline in Full Analysis Set, N | 58 | 59 | 117 |
| Age, years |  |  |  |
| Mean (SD) | 40.5 (13.27) | 43.7 (13.27) | 42.1 (13.31) |
| Sex |  |  |  |
| Female, n (%) | 32 (55.2) | 37 (62.7) | 69 (59.0) |
| Age at diagnosis |  |  |  |
| Mean (SD) | 25.9 (13.34) | 28.2 (12.81) | 27.0 (13.07) |
| Crohn’s Disease duration, years |  |  |  |
| Mean (SD) | 14.6 (10.41) | 15.5 (11.04) | 15.1 (10.70) |
| Involved GI areas, n (%) |  |  |  |
| Ileum only | 14 (24.1) | 16 (27.1) | 30 (25.6) |
| Colon only | 14 (24.1) | 15 (25.4) | 29 (24.8) |
| Ileum and Colon | 29 (50.0) | 28 (47.5) | 57 (48.7) |
| Proximal | 4 (6.9) | 9 (15.3) | 13 (11.1) |
| Perianal | 14 (24.1) | 19 (32.2) | 33 (28.2) |
| Patients with MTX/AZA/6-MP or corticosteroid use at baseline | 22 (37.9%) | 19 (32.2%) | 41 (35.0%) |
| Immunomodulators (MTX/AZA/6-MP) | 10 (17.2%) | 10 (16.9%) | 20 (17.1%) |
| Corticosteroids (including budesonide) | 13 (22.4%) | 11 (18.6%) | 24 (20.5%) |
| Corticosteroids (excluding budesonide) | 7 (12.1%) | 6 (10.2%) | 13 (11.1%) |
| Patients with inadequate response to corticosteroids and MTX/AZA/6-MP | 47 (81.0%) | 51 (86.4%) | 98 (83.8%) |
| Patients with inadequate response to corticosteroids only | 7 (12.1%) | 7 (11.9%) | 14 (12.0%) |
| Patients with inadequate response to MTX/AZA/6-MP only | 15 (25.9%) | 14 (23.7%) | 29 (24.8%) |
| History of inadequate response or intolerance to biologics before ustekinumab, ^a,b^ n (%) |  |  |  |
| No history of inadequate response or intolerance (biologic naive or experienced) | 4 (6.9%) | 6 (10.2%) | 10 (8.5%) |
| ≥1 biologic (anti-TNF agent or vedolizumab) before ustekinumab, ^a,b^ | 54 (93.1%) | 53 (89.8%) | 107 (91.5%) |
| ≥2 biologics (≥1 anti-TNF agents ± vedolizumab) before ustekinumab, ^a,b^ | 31 (53.4%) | 36 (61.0%) | 67 (57.3%) |
| ≥3 biologics (1 anti-TNF agent ± vedolizumab before ustekinumab, ^a,b^ | 17 (29.3%) | 19 (32.2%) | 36 (30.8%) |
| ≥1 anti-TNF + vedolizumab^c^ before ustekinumab, ^a,b^ | 21 (36.2%) | 20 (33.9%) | 41 (35.0%) |
| CDAI score |  |  |  |
| Mean (SD) | 288.1 (54.95) | 285.2 (58.18) | 286.6 (56.38) |
| PRO-2 (without weighting)^d^ |  |  |  |
| Median | 47.5 | 48.0 | N/A |
| IQ range | (36.0; 59.0) | (35.0; 62.0) | N/A |
| CRP (mg/L) |  |  |  |
| Median | 4.9 | 5.4 | 5.0 |
| IQ range | (2.5; 13.0) | (2.2; 12.4) | (2.3; 12.4) |
| fCal (mg/kg) |  |  |  |
| Median | 642.0 | 484.0 | 515.0 |
| IQ range | (126.0; 2140.0) | (167.0; 1516.0) | (161.0; 1735.0) |
| IBDQ score^e^ (32-224) |  |  |  |
| N | 56 | 59 | 115 |
| Mean (SD) | 119.5 (33.86) | 116.2 (27.14) | 117.8 (30.51) |
| SES-CD score^f^ |  |  |  |
| Mean (SD) | 11.5 (7.00) | 10.4 (6.75) | 10.9 (6.86) |

CDAI, Crohn’s Disease Activity Index; CRP, C-reactive protein; fCal, fecal calprotectin; GI, gastrointestinal; IBDQ, Inflammatory Bowel Disease Questionnaire; IQ, interquartile; PRO-2, Patient Reported Outcome; IV, intravenous; SC, subcutaneous; SD, standard deviation; SES-CD, Simple Endoscopic Score for Crohn’s Disease; TNF, tumor necrosis factor

^a^ Patients with a history inadequate response or intolerance to biologic treatment characterized by primary nonresponse, secondary nonresponse, or intolerance.

^b^ Anti-TNFs were adalimumab, infliximab, and certolizumab pegol.

^c^ Patients with inadequate response or intolerance to at least 2 mechanisms of action before receiving ustekinumab, regardless of the number of anti-TNFs.

^d^ Sum of the number of stools and the abdominal pain scores in the previous 7 days.

^e^ The IBDQ total scores were used, ranging from 32 to 224.

^f^ Endoscopy was an optional procedure during the study.

**Supplemental Figure 1.** **Pharmacokinetic modelling of ~6mg/kg IV Reinduction Versus SC 90 mg q4w Injections**


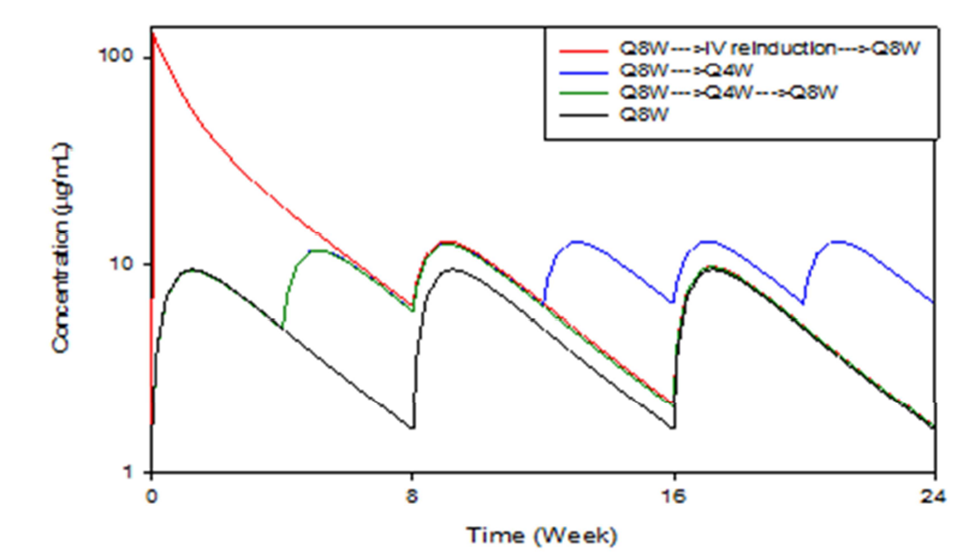


IV, intravenous, q4w, every 4 weeks; q8w, every 8 weeks; SC, subcutaneous

**Supplemental Figure 2. POWER Study Design**

BW, body weight; CD, Crohn’s disease; CDAI, Crohn’s Disease Activity Index; CRP c-reactive protein; fCal, fecal calprotectin; IV, intravenous; LoR, loss of response; PBO, placebo; q8w, every 8 weeks; R, randomization.SC, subcutaneous; UST, ustekinumab

^a^ Loss of response was defined by baseline CDAI of ≥ 220–450 and at least 1 of the following: elevated CRP (> 3.0 mg/L), elevated fCal (> 250 mg/kg), or endoscopy (performed within the 3 months before baseline) with evidence of active CD during the current disease flare (i.e. ≥ 1 ulcerations in the ileum and/or colon).

^b^ Clinical response was defined as a decrease of ≥ 100 points from Week 0 or CDAI score of < 150 points.

**Supplemental Figure 3. CONSORT Diagram**

IV, intravenous; SC, subcutaneous

**Supplemental Figure 4. IBDQ Response and Remission at Week 16**

CDAI, Crohn’s Disease Activity Index; CI, confidence interval; IBDQ, Inflammatory Bowel Disease Questionnaire; IV, intravenous; SC, subcutaneous; UST, ustekinumab

As the primary endpoint was not met, all p values for other comparisons between treatment groups should be considered nominal. IBDQ response was defined as a ≥16-point improvement from baseline and IBDQ remission was defined as an IBDQ score ≥170 ^b,c^

^a^ 2‑sided Cochran–Mantel–Haenszel–chi-square test, stratified by baseline CDAI score (≤ 300 or > 300) and prior biologic failure status at baseline (yes or no) at a significance level of 0.05; the 95% CIs were based on the Wald statistic with Mantel–Haenszel weight.

^b^ Patients who had a prohibited Crohn's disease-related surgery, had prohibited concomitant medication changes, or discontinued study agent due to lack of efficacy or due to an adverse event indicated to be of worsening Crohn's disease prior to the designated analysis timepoint are considered not to have achieved IBDQ response/remission.

^c^ Patients who had insufficient data at the designated analysis timepoint are considered not to have achieved IBDQ response/remission.

**Supplemental Figure 5. Clinical Response at Week 16 by Baseline Characteristics**

**A.**

**B.**

6-MP, 6-mercaptopurine; AZA, azathioprine; CD, Crohn’ disease; CDAI, Crohn’s Disease Activity Index; CI, confidence interval; CRP, C-Reactive Protein; fCal, fecal calprotectin; GI, gastrointestinal; IV, intravenous; MTX, methotrexate; NE, not estimable; OR, odds ratio; q8w, every 8 weeks; SC, subcutaneous; SES-CD, Simple Endoscopic Score for Crohn’s Disease; UST, ustekinumab

**Supplemental Figure 6. Median Serum Ustekinumab Concentrations**

**
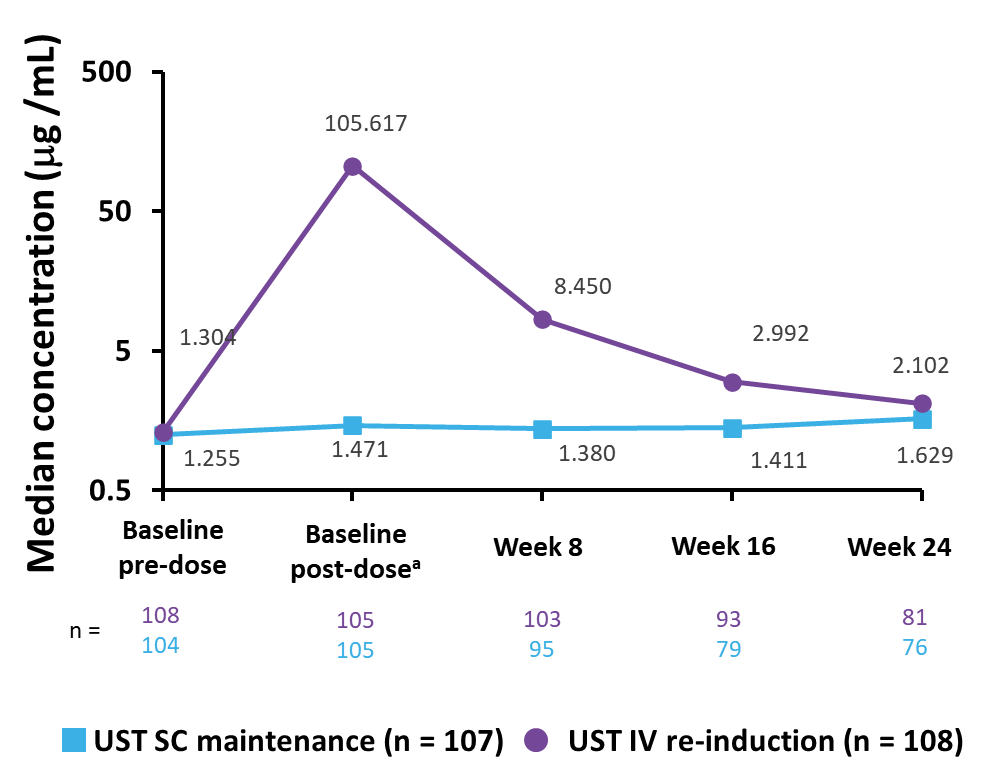
**

IV, intravenous; SC, subcutaneous; UST; ustekinumab

^a^ The post-dose sample was collected within 24 hours after the administration of ustekinumab.

**Supplemental Figure 7.** **Patients in Clinical Response at Week 16 by Ustekinumab Serum Trough Concentration Quartile Groups at Baseline ^a, c, d^**

**
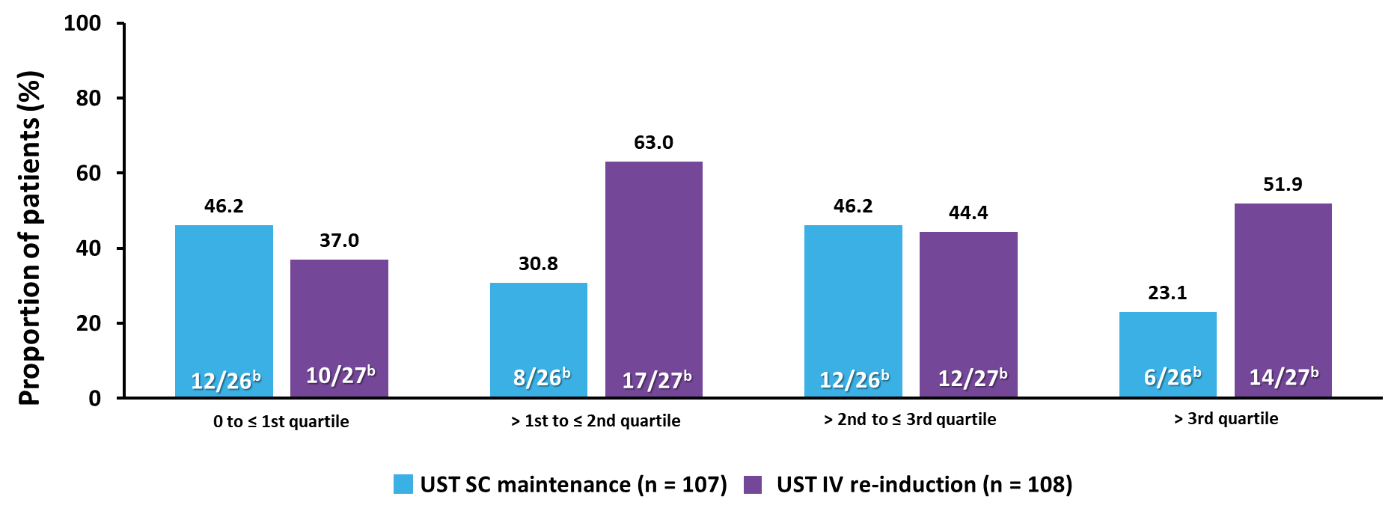
**

CDAI, Crohn’s Disease Activity Index; IV, intravenous; SC, subcutaneous; UST, ustekinumab

^a^ Quartiles are based on patients in each treatment group as follows: Ustekinumab SC: 1st quartile = 0.57 µg/mL, 2nd quartile = 1.26 µg/mL, 3rd quartile = 2.53 µg/mL. Ustekinumab IV: 1st quartile = 0.62 µg/mL, 2nd quartile = 1.30 µg/mL, 3rd quartile = 2.79 µg/mL.
 ^b^ Number of patients with serum concentration in the designated category.
 ^c^ Patients who had a prohibited Crohn's disease-related surgery, had prohibited concomitant medication changes, or discontinued study agent due to lack of efficacy or due to an adverse event indicated to be of worsening Crohn's disease prior to the designated analysis timepoint are considered not to be in clinical response, regardless of their CDAI score.
 ^d^ Patients who had insufficient data to calculate the CDAI score at the designated analysis timepoint are considered not to be in clinical response.

**Supplemental Figure 8. Endoscopic Remission at Week 16 by (A) Ustekinumab Serum Trough Concentration and (B) Ustekinumab Trough Quartiles ^a,b^**

1. **Ustekinumab Trough Concentration at Week 16**

**
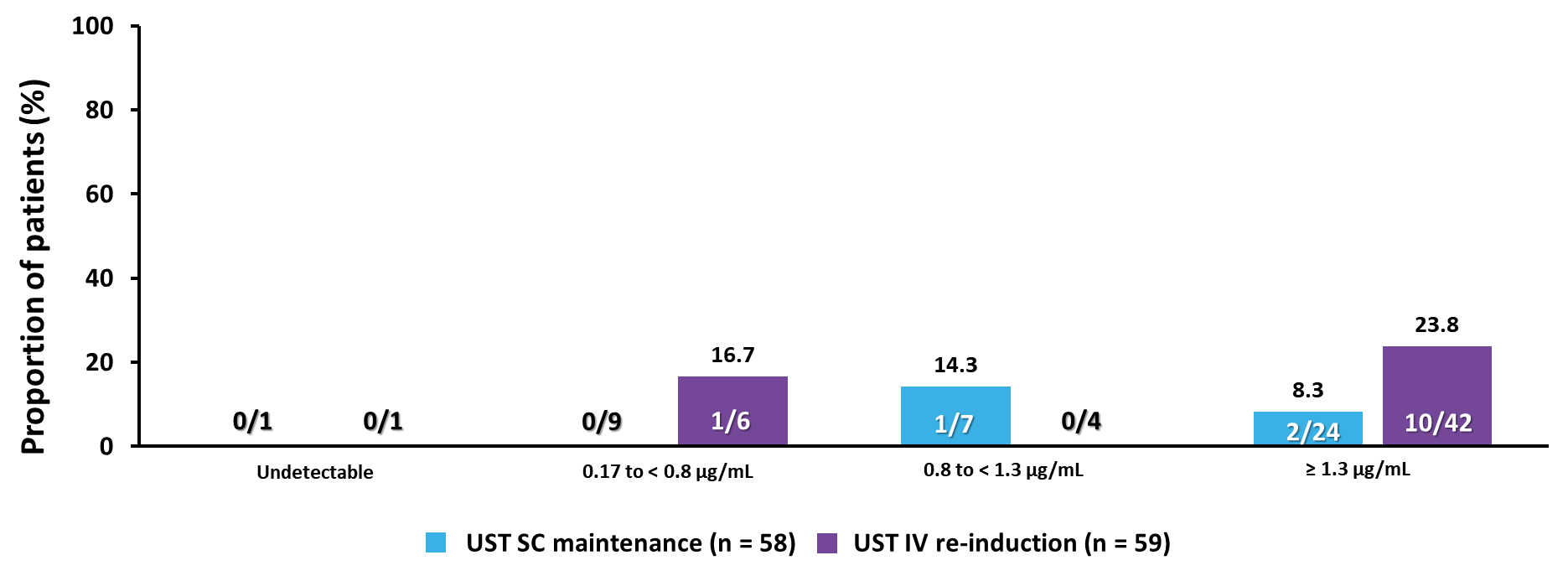
**

1. **Ustekinumab Trough Concentration Quartiles at Week 16**

**
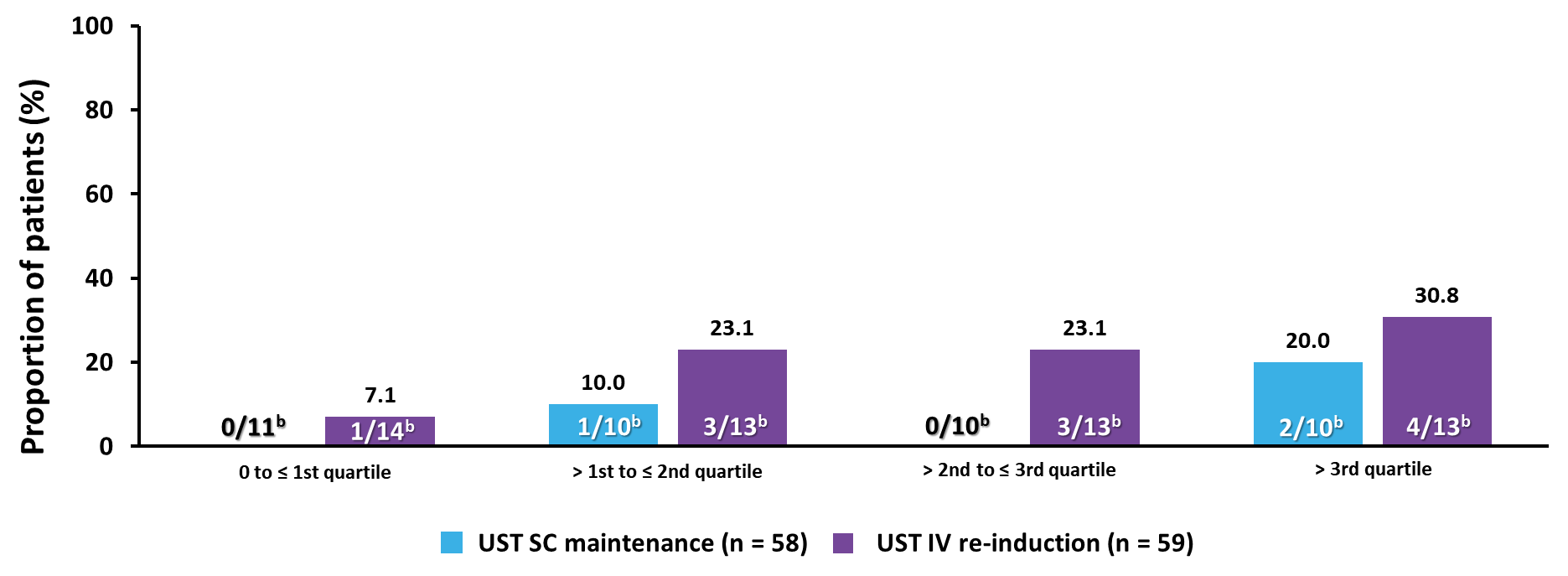
**

IV, intravenous; SC, subcutaneous; UST, ustekinumab

Endoscopic remission is defined as Simple Endoscopic Score for Crohn’s disease (SES-CD) score ≤3 or SES-CD=0 for patients who enter the study with an SES-CD=3.

^a^ Patients who had a prohibited Crohn's disease-related surgery, discontinued due to lack of efficacy or due to an adverse event indicated to be of worsening Crohn's disease, or had prohibited concomitant medication changes prior to the designated analysis time point are considered not to be in endoscopic remission, regardless of their SES-CD score.

^b^ Patients who had insufficient data to calculate the CDAI score at the designated analysis timepoint were considered not to be in endoscopic remission.

Quartiles are based on subjects with both available pharmacokinetic sample and baseline SES-CD score ≥3 in each treatment group as follows: Ustekinumab SC: 1st quartile = 0.82 µg/mL, 2nd quartile = 1.41 µg/mL, 3rd quartile = 2.49 µg/mL. Ustekinumab IV: 1st quartile = 1.67 µg/mL, 2nd quartile = 3.60 µg/mL, 3rd quartile = 5.39 µg/mL.
